# Synthesis of a Ni Complex Chelated by a [2.2]Paracyclophane-Functionalized Diimine Ligand and Its Catalytic Activity for Olefin Oligomerization

**DOI:** 10.3390/molecules26092719

**Published:** 2021-05-05

**Authors:** Daisuke Takeuchi, Yoshi-aki Tojo, Kohtaro Osakada

**Affiliations:** 1 Laboratory for Chemistry and Life Science, Tokyo Institute of Technology, 4259 Nagatsuta, Midori-ku, Yokohama 226-8503, Japan; tojo.yoshiaki.ma@m-chemical.co.jp; 2 Department of Frontier Materials Chemistry, Faculty of Science and Technology, Hirosaki University, 3 Bunkyo-cho, Hirosaki-shi, Aomori 036-8561, Japan; 3 National Institute of Advanced Industrial Science and Technology (AIST), Tsukuba Central 5, 1-1-1 Higashi, Tsukuba, Ibaraki 305-8565, Japan

**Keywords:** oligomerization, olefin, nickel, catalysts, *N*-ligand

## Abstract

A diimine ligand having two [2.2]paracyclophanyl substituents at the N atoms (**L1**) was prepared from the reaction of amino[2.2]paracyclophane with acenaphtenequinone. The ligand reacts with NiBr_2_(dme) (dme: 1,2-dimethoxyethane) to form the dibromonickel complex with (*R*,*R*) and (*S*,*S*) configuration, NiBr_2_(**L1**). The structure of the complex was confirmed by X-ray crystallography. NiBr_2_(**L1**) catalyzes oligomerization of ethylene in the presence of methylaluminoxane (MAO) co-catalyst at 10–50 °C to form a mixture of 1- and 2-butenes after 3 h. The reactions for 6 h and 8 h at 25 °C causes further increase of 2-butene formed via isomerization of 1-butene and formation of hexenes. Reaction of 1-hexene catalyzed by NiBr_2_(**L1**)–MAO produces 2-hexene via isomerization and C12 and C18 hydrocarbons via oligomerization. Consumption of 1-hexene of the reaction obeys first-order kinetics. The kinetic parameters were obtained to be Δ*G*^‡^ = 93.6 kJ mol^−1^, Δ*H*^‡^ = 63.0 kJ mol^−1^, and Δ*S*^‡^ = −112 J mol^−1^deg^−1^. NiBr_2_(**L1**) catalyzes co-dimerization of ethylene and 1-hexene to form C8 hydrocarbons with higher rate and selectivity than the tetramerization of ethylene.

## 1. Introduction

The oligomerization of olefins catalyzed by transition metal complexes has attracted attention, as shown by many review articles on this topic over the last decades [1,2,3,4,5,6,7,8,9,10,11,12,13,14,15,16] as well as recent original reports [17,18,19]. It is related to the industrial production of unsaturated hydrocarbon materials. The mechanistic studies are of interest from the viewpoint of catalytic and organometallic chemistry. Various complexes of early and late transition metals are employed as the catalyst for the oligomerization. Transition metal complexes were reported to promote cross-dimerization of two alkynes and of alkyne with vinyl compounds to form enynes and dienes, respectively [20,21,22,23,24]. The cross-dimerization of two vinyl compounds has been focused on hydrovinylation of styrene and of olefins containing polar fnctional groups [25,26,27,28,29,30,31,32,33,34,35]. α,ω-Dienes undergo transition metal–catalyzed intramolecular hydrovinylation, which provides a convenient route to the cycloolefins [36,37,38,39]. On the other hand, intermolecular cross-dimerization of two hydrocarbon alkenes is rare. Hessen reported that a constrained geometry complex (CGC)-type Ti complex catalyzed cross-trimerization of ethylene with 1–octene to form C12 products [40].

Ni and Pd complexes with diimine ligands having bulky *N*-aryl substituents were found to catalyze high-mass polymerization of ethylene and 1-olefins as well as copolymerization of ethylene with acrylates [41]. The complexes with 2,6-disubstituted aryl groups at the coordinating nitrogens, **1a**–**1f**, catalyze ethylene polymerization. Complexes **1g** and **1h** with 4-substituted aryl groups at the nitrogen atoms catalyze oligomerization of ethylene to form α-olefins with Schultz–Flory distribution [42,43]. Subsequent studies using Ni and Pd complexes with strically bulky diimine ligands, **1i**–**1p**, as the catalysts revealed the polymerization and co-polymerization of olefins with high productivity and selectivity [44,45,46,47,48,49,50,51,52,53,54,55,56].

**Scheme 1:**
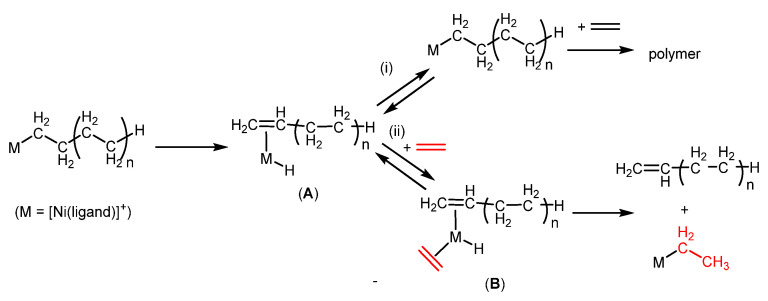
Polymerization vs. oligomerization. (**A**,**B**) denote intermediates of the reactions.

Occurrence of polymerization or oligomerization of ethylene depending on the substituents of the diimine ligand is rationalized by the insertion–β-hydrogen elimination mechanism, as shown in Scheme 1. The growing polymer having an alkyl–nickel bond undergoes β-hydrogen elimination of vinyl group-terminated oligomer to form a hydride(olefin)nickel(II) species (**A**).

Intermediate (**A**) with the ligand having 2,6-disubstituted *N*-aryl groups prefers re-insertion of the vinyl group into the Ni–H bond, and resumes the polymer growth (path (i)). Ni center of intermediate (**A**) having the diimine ligand with 4-substituted *N*-aryl groups is sterically less crowded, and undergoes associative coorindation of an ethylene monomer at the apical coordination site of square-planar Ni(II) center, forming intermediate (**B**) (path (ii)). The reaction is followed by elimination of the oligomer having a vinyl end group and insertion of ethylene into the H–Ni bond. Further insertion of ethylene molecules into the Ni–C bond provides new oligomer molecules. In this study, we synthesized the Ni complex with a diimine ligand having [2.2]paracyclophanyl substituents at the *N*-positions. The complex is expected to show new catalytic properties because of the sterically bulky *N*-cycloparaphenyl groups of the ligand. It catalyzes olefin oligomerization, and ethylene–1-hexene co-dimerization, in particular. Here, we report synthesis and structure of the new Ni-diimine complexes as well as its catalysis.

## 2. Results and Discussion

### 2.1. Preparation and Structure of Ni Complexes

Mono-substituted [2.2]paracyclophane has a double-decker structure with a chiral center in the molecule. The transition metal complexes with the paracyclophane-containing nitrogen ligand, such as a Ti-Salen complex [57] and Au and Rh complexes with *N*-heterocyclic carbene (NHC) ligands [58,59,60], were employed as the catalyst for stereoselective reactions. We conducted condensation of acenaphtenequinone with two molar equivalents of amino[2.2]paracyclophane with expecting formation of a diimine ligand having two [2.2]paracyclophanyl substituents. The reaction in refluxing EtOH-AcOH proceeds smoothly to form the ligand, according to Equation (1). Both racemic and optically active amino[2.2]paracyclophanes were used in the ligand synthesis.


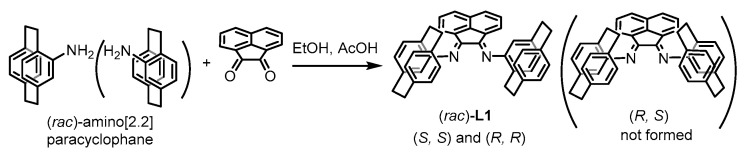
(1)

**Figure 1:**
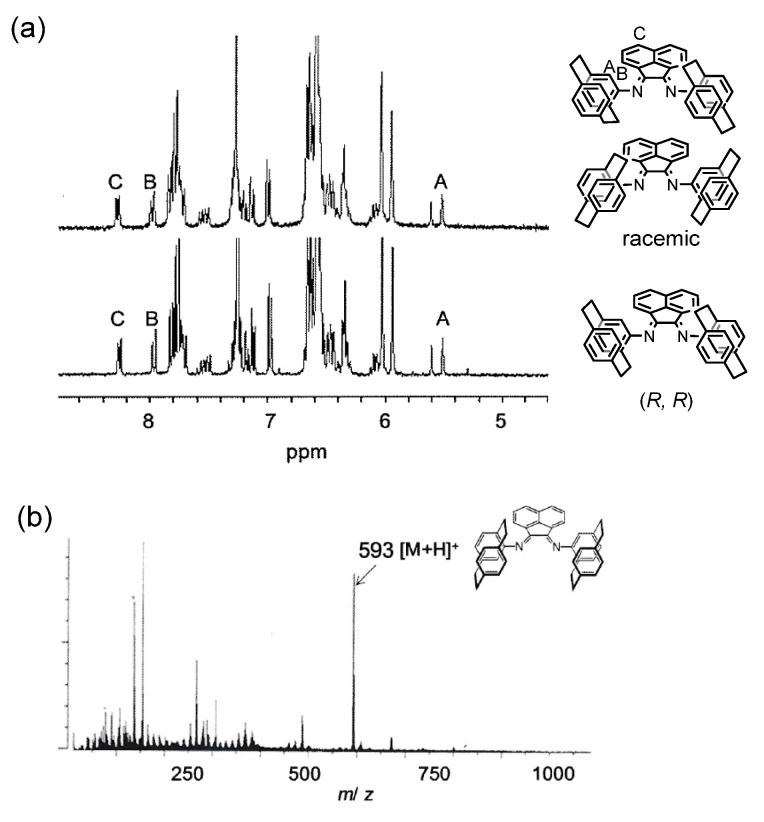
(**a**) ^1^H NMR spectra (aromatic hydrogen region) of ligand **L1** obtained from racemic amino[2.2]paracyclophane (upper) and from the optically amino[2.2]paracyclophane (lower). (**b**) FAB-MS spectrum of racemic **L1** obtained by using 2-nitrobenzyl alcohol as the matrix.

Figure 1a shows the ^1^H NMR spectra of the ligand **L1**, obtained from the racemic (upper) and optically active (lower) amino[2.2]paracyclophanes, respectively. The characteristic aromatic hydrogen signals near the imine group are observed at the same positions. Total spectra of the ligand from the racemic and optically active starting materials are also identical. It suggests that the ligand from a racemic mixture has (*R*,*R*) or (*S*,*S*) configuration. The ligand having a meso structure with (*R*, *S*) or (*S*, *R*) configuration is not contained in the product. Figure 1b shows results of FAB-MAS measurement of **L1**. The parent peak at *m*/*z* = 593 corresponds to [M–H]^+^ of **L1**. These spectroscopic data as well as the results of elemental analysis clearly indicate the formation of ligand **L1** in a pure form. Thus, condensation of acenaphtenequinone with racemic amino[2.2]paracyclophane forms **L1** diastereoselectively. We used the ligand obtained from racemic amino[2.2]paracyclophane for prepartion of the catalysts of this study.

**Figure 2:**
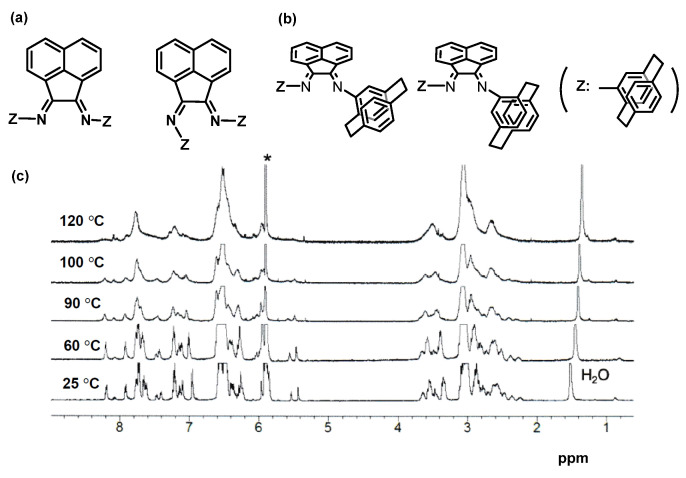
(**a**,**b**) Possible conformational isomers of **L1**. (**c**) Temperature dependent ^1^H NMR spectra of **L1** at 25–120 °C.

The above ^1^H NMR spectra of **L1** in Figure 1a contains the signals with a more number than that expected from the molecular structure. It is attributed to the presence of conformational isomers of the compounds in the solution. Figure 2a depicts two isomers due to *E* and *Z* geometry about the C=N bond, while Figure 2b shows possible isomers by rotation of the C-N bond between the [2.2]paracyclophanyl group and the imine group. Sterically crowded structure of the molecule renders interconversion of the isomers difficult even in the solution. Figure 2c shows the ^1^H NMR spectra at high temperatures. The signals are broadened above 90 °C, but do not undergo coalescence, which suggests that the interconversion among the conformational isomers is slower than the NMR time scale.

Ligand **L1** reacts with NiBr_2_(dme) (dme = 1,2-dimethoxyethane) at room temperature to form the complex formulated as NiBr_2_(**L1**), as shown in Equation (2). A direct reaction of NiBr_2_ with 2,5-dimethylaniline and acetonaphtequinone produces Ni compex with a ligand having 2,5-dimethylphenyl substituents at the imine nitrogen, NiBr_2_(**L2**), as shown in Equation (3). Ligand **L2** also has 2,5-disubstituted aryl groups at the imine nitrogens, similar to **L1**, but is sterically much less bulky than **L1**. Catalytic activity of the complex is compared with that of NiBr_2_(**L1**), having the sterically more crowded ligand.


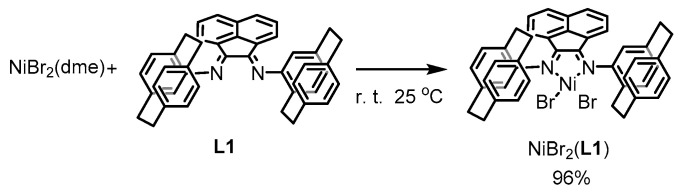
(2)


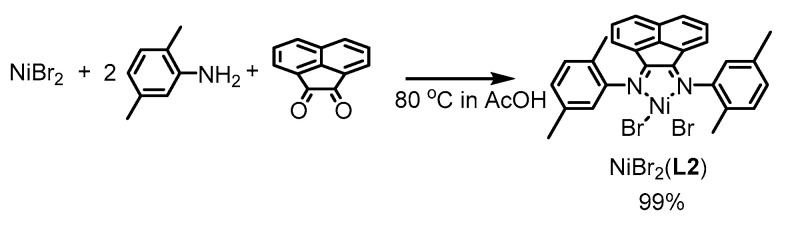
(3)

**Figure 3:**
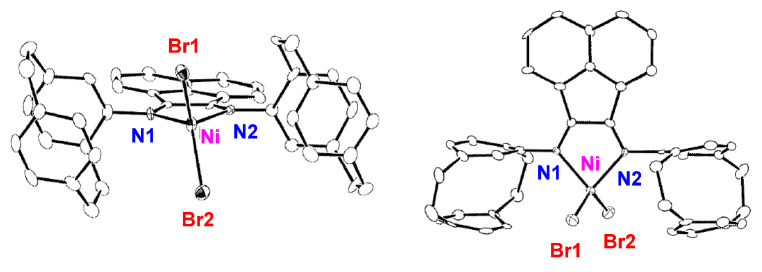
Crystallographic structure of NiBr_2_(**L1**)·(C_2_H_4_Cl_2_). Selected bond distances (Å) and angles (°): Ni1–Br1 2.3375(15), Ni1–Br2 2.3392(16), Ni1–N1 2.028(5), Ni1–N2 2.035(5), Br1–Ni1–Br2 128.90(5), Br1–Ni1–N1 104.45(15), Br1–Ni1–N2 113.19(15), Br2–Ni1–N2 104.02(15), Br2–Ni1–N1 113.54(15), N1–Ni1–N2 83.7(2). The solvent molecule and hydrogen atoms are omitted for simplicity.

Figure 3 shows the molecular structure of NiBr_2_(**L1**) determined by X-ray crystallography [61]. Two [2.2]paracyclophanyl substituents are orientated to the opposite side of the acenaphtene group. The Ni center has the distorted tetrahedral structure, suggesting paramagnetic high-spin complex of a *d*^8^ metal center. The [2.2]paracyclophanyl substituents of the ligand are expected to influence stability of the intermediates with polymer and monomer ligands and selectivity of the reaction.

**Chart 1:**
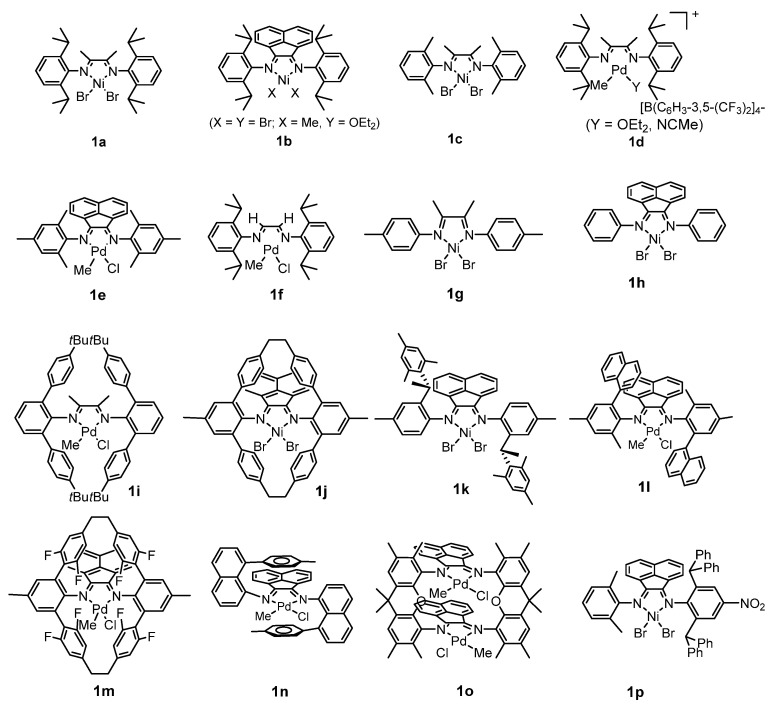
Ni and Pd diimine complexes for catalytic polymerization and oligomerization.

The crystal structure indicates that the ligand and Ni center forms a *C*_2_ symmetrical space around the Ni center. Ni and Pd complexes **1k**, **1l**, **1n** in Chart 1 also have coordination of the diimine ligand with *C*_2_ symmetrical structures. Polymerization of olefins using these complexes as the catalyst was reported to occur stereoelectively. Investigation of a dinickel catalyst having a *C*_2_ symmetrical space around the Ni(II) center revealed relevance of the detailed coordination structure of the complex to productivity and selectivity of the catalysis [62].

### 2.2. Olefin Oligomerization Catalyzed by Ni Complexes

Oligomerization of ethylene and 1-hexene was studied by using NiBr_2_(**L1**) as the catalyst and methylaluminoxane (MAO) as the co-catalyst. Table 1 summarizes results of ethylene oligomerization catalyzed by NiBr_2_(**L1**). The reactions at 10 °C form mixtures of 1-butene and 2-butene (entries 1, 2). The reactions at 25 °C form the butenes in larger amounts and C6 hydrocarbon products, as confirmed by GPC analysis (entries 3–6).

**Figure 4:**
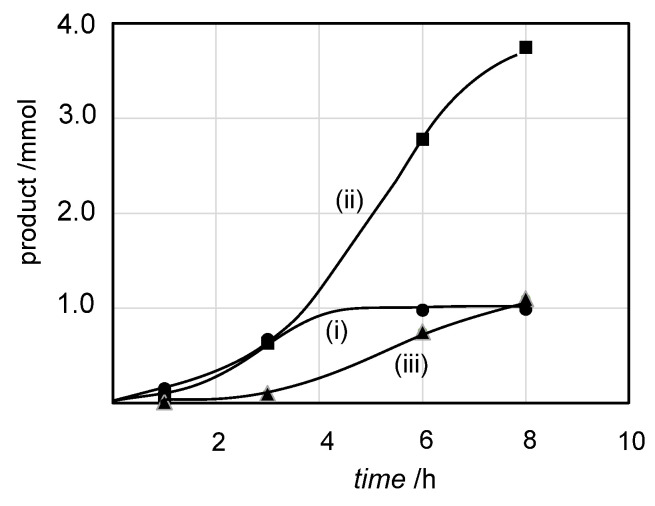
Reaction profile of ethylene oligomerization catalyzed by NiBr_2_(**L1**)–MAO; (i) 1-butene, (ii) 2-butene, (iii) 1-hexene. Ethylene: 1 atm, Ni: 0.010 mol, [Al]/[Ni] = 300, toluene 10 mL, 25 °C. Product amounts are determined by GPC analysis.

The reaction yields 1- and 2-butenes in 3:1 molar ratio after 1 h, while further reaction causes relative increase of 2-butene and formation of hexenes after 3 h. Figure 4 plots time profile of the reaction, which suggests that initially formed 1-butene is isomerized into 2-butene during the reaction. Turn over frequency (TOF) for formation of the butenes increases for initial 6 h, and becomes constant after 6 h. It suggests that active species of the catalysis are increased slowly under the conditions.

Maximum TOF of the reaction is calculated from the total amount of 1- and 2-butenes to be 124–126 (h^−1^) under 1 atm ethylene at 25 °C (entries 5, 6). Ni-diimine complex with 4-methylphenyl substituents at the imine nitrogen, **1g**, was reported to catalyze ethylene oligomerization to α-olefins up to C20 with TOF of 53,000–57,000 (h^−1^) at 35 °C under 56 atm of ethylene [42]. TOF of the reaction catalyzed by NiBr_2_(**L1**) and averaged carbon number of the products are smaller than **1g**, even when different temperature and ethylene pressure are considered. It is ascribed to severe steric hindrance of the Ni center of NiBr_2_(**L1**) bonded with the diimine ligand with [2.2]paracyclophane substituents. Reaction of ethylene catalyzed by NiBr_2_(**L2**) under similar conditions did not form C4- nor C6- oligomers, but produced a low molecular weight polyethylene as a wax solid (*M*_n_ = 1000, *M*_w_/*M*_n_ = 2.87 based on GPC using polystyrene standards) (entry 7). The activity of the reaction by NiBr_2_(**L1**) catalyst at 50 °C is much lower than 25 °C (entries 8,9).

The catalytic activity of NiBr_2_(**L1**) is compared with the Ni-diimine complexes reported so far. The Ni complex having 4-alkylphenyl groups at the imine nitrogen of the diamine ligand catalyzes ethylene oligomerization with high TOF because of frequent β-hydrogen elimination of the oligomers caused by associative exchange of the coordinated oligomer molecule by a new ethylene monomer [42]. The complex with 2,5-disubstituted phenyl group, NiBr_2_(**L2**), also produces the oligomer with *M*_n_ = 1000, as shown above. The complexes having bulky 2,6-disubstituted or 2,4,6-trisubstituted aryl groups at the diimine nitrogen catalyze high mass polymerization of ethylene because the associative chain transfer of the polymer molecule is inhibited strictly by the bulky aryl groups at the imine nitrogen [41]. NiBr_2_(**L1**) of this study has a more bulky ligand than the ligands of the above studies, and catalyzes dimerization and trimerization of ethylene.

**Figure 5:**
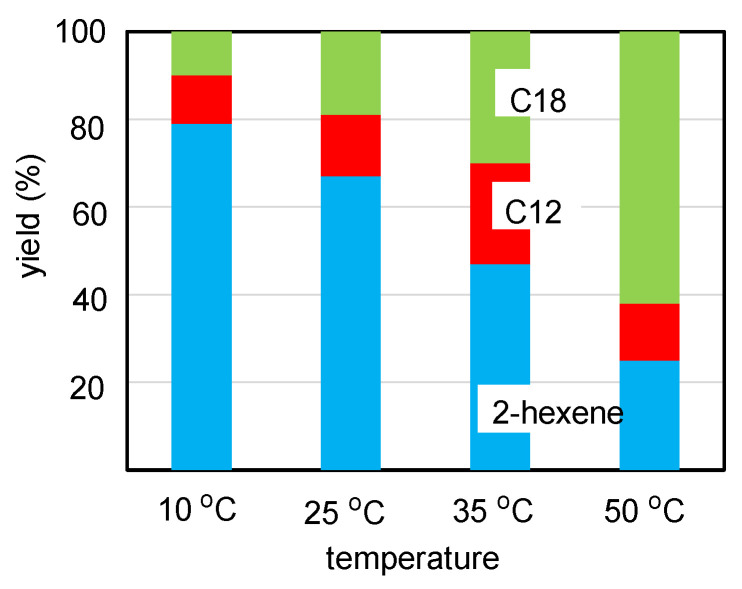
Temperature effect of oligomerization of 1-hexene. 1-Hexene: 3.0 mmol, Ni: 0.010 mol, [Al]/[Ni] = 300, toluene 10 mL, 24 h. Conversion of 1-hexene is 83–95% for the reactions.

Reaction of 1-hexene catalyzed by NiBr_2_(**L1**)-MAO ([Al]/[Ni] = 300) causes isomerization of the substrate to 2-hexene and dimerization and trimerization of 1-hexene to form C12 and C18 products. The isomerization occurs more readily than the oligomerization under the examined conditions. Results of the reactions under different conditions are summarized in Table 2. The reactions at 10 °C with MAO ([Al]/[Ni] = 300) and at 25 °C with a smaller amount of MAO ([Al]/[Ni] = 50) (entries 1–3) show lower catalytic activity than those at 25 °C and [Al]/[Ni] = 300 (entry 4, 5). At 35 °C and 50 °C, TOF for the oligomerization is high for the initial 0.5 h (28 and 61/h^−1^, respectively) and become much lower after 6 h. It indicates that the catalytic activity decreases rapidly for several hours. The addition of MAO in a larger amount ([Al]/[Ni] = 1000) does not increase the oligomer yields. The product ratios after the reaction for 24 h vary depending on the temperature (entries 2, 5, 9, 12), which is shown in Figure 5. The reaction for 24 h at 50 °C forms the trimer as the main product (entry 12). Use of modified methylaluminoxane (MMAO) as the co-catalyst decreases the oligomer yields (entry 13). The reactions using AlMe_3_ and Et_2_AlCl co-catalysts yield 2-hexene exclusively (entries 14, 15).

**Figure 6:**
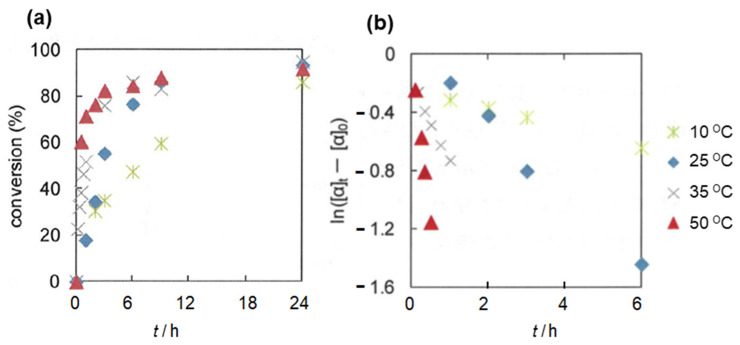
Oligomerization of 1-hexene catalyzed by NiBr_2_(**L1**)–MAO. (**a**) Time conversion of 1-hexene at 25 °C. (**b**) First-order plots of the total reaction.

Figure 6 shows time-conversion (a) and first-order plots (b) of the total reaction at 10 °C, 25 °C, 35 °C, and 50 °C. The reaction obeys first-order kinetics to the concentration of 1-hexene. The kinetic parameters of the reaction were determined from Eyring plots to be Δ*G*^‡^ = 93.6 kJ mol^−1^, Δ*H*^‡^ = 63.0 kJ mol^−1^, Δ*S*^‡^ = −112 J mol^−1^deg^−1^. Isomerization of 1-hexene into 2-hexene proceeds via insertion of the olefin into a Ni–H bond and subsequent β-hydrogen elimination of the internal olefin. Formation of C12 and C18 products is induced by insertion of 1-hexene into the Ni-C bond followed by β-hydrogen elimination of the products. The above kinetics for the reaction suggests that insertion of 1-hexene into the Ni–H and Ni–C bonds is the rate-determining step of the reaction.

**Figure 7:**
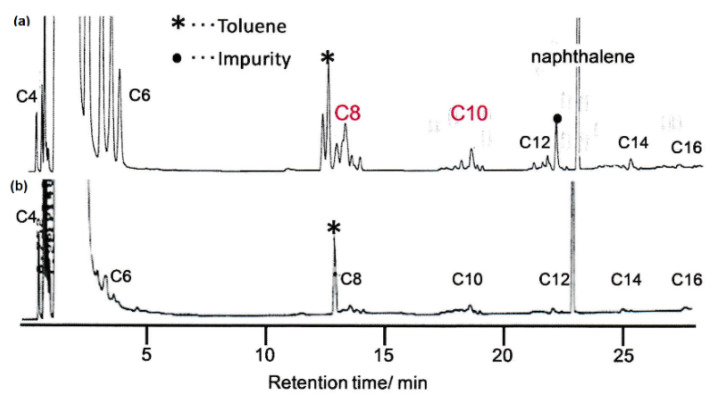
GC elution of the product. (**a**) Reaction of ethylene and 1-hexene catalyzed by NiBr_2_(**L1**) −MMAO. (**b**) Reaction of ethylene catalyzed by NiBr_2_(**L1**) −MMAO. Ni complex 0.010 mmol, [Al]/[Ni] = 300. Pentane 8 mL, 25 °C, 24 h.

Reaction of a mixture of ethylene and 1-hexene catalyzed byNiBr_2_(**L1**)-MMAO formed C8 products in a higher amount than C10–C16 products. Figure 7 compares results of GLC measurement of the reaction mixture with that of ethylene oligomerization under similar conditions. The products of the reaction of 1-hexene under ethylene atmosphere contain C8 (0.92 mmol), C10 (0.24 mmol), and C12 (0.095 mmol), as shown in Figure 7a. Figure 7b shows the results of the reaction of ethylene, producing C4 and C6 hydrocarbons in main. The amounts of higher hydrocarbon products, C8 (0.076 mol), C10 (0.046 mmol), and C12 (0.017 mmol), are smaller than the reaction of ethylene and 1-hexene, as shown in Figure 7b. Thus, the reaction of ethylene and 1-hexene forms the hydrocarbon via cross-dimerization much more rapidly than tetramerization of ethylene and cross-trimerization (C10 hydrocarbons), and cross-tetramerization (C12 hydrocarbons). The experimental results at present, however, are not sufficient to discuss detailed reaction pathways for the selective cross-dimerization.

## 3. Conclusions

This paper presents diastereoselective preparation of dimine ligand **L1** with two [2.2]paracyclophanyl groups, via condensation of acenaphtenequinone with two equivalents of amino[2.2]paracyclopheylene, and its complexation with Ni(II) center to form NiBr_2_(**L1**). X-ray crystallographic results of NiBr_2_(**L1**) showed the molecular structure whose paracyclophanyl groups are at the positions close to the Ni center. The complex, in the presence of MAO co-catalyst, catalyzes oligomerization of ethylene to form mixtures of 1- and 2-butenes at 10–50 °C with the highest TOF for butene formation (126 h^−^^1^). The reaction of 1-hexene using the same catalyst causes isomerization into 2-hexene and oligomerization to C12 and C18 products. The total reaction obeys first-order kinetics to the amount of 1-hexene, suggesting the rate-determining step at the insertion of 1-hexene into Ni–H and Ni–C bonds. NiBr_2_(**L1**) catalyzes cross-dimerization of ethylene with 1-hexene to form C8 products in the presence of MMAO, which occurs more readily than tetramerization of ethylene and than the cross-oligomerization of the two olefins, giving C10 and C12 products, under the same conditions. Thus, NiBr_2_(**L1**) with an extremely bulky diimine ligand catalyze dimerization and trimerization of ethylene rather than formation of higher oligomers or high mass polymers. The unique properties of the catalysis is a selective formation of the cross-dimer of ethylene and 1-hexene. The elucidation of the mechanism for the selective co-dimerization reaction is a problem left for future research.

## 4. Experimental Section

### 4.1. General

All the chemicals were commercially available. MAO and MMAO were purchased from Tosoh Co. Ltd. (Tokyo, Japan) as toluene solutions. ^1^H and ^13^C{^1^H} NMR spectra were acquired on a Bruker AV-400M. The chemical shifts were referenced with respect to CHCl_3_ (δ 7.26), HDO (δ 4.79) for ^1^H, and CDCl_3_ (δ 77.0), DSS (sodium 3-(trimethylsilyl)-1-propanesulfonate) (δ 0.0) for ^13^C as internal standards.

### 4.2. Preparation of Racemic Ligand ***L1***

(*rac*)-Amino[2.2]paracylophane was prepared by the reported method [63], and modification of the final step, Curtius rearrangement, results in the product in overall 61% yield. A mixture of *rac*-amino[2.2]paracyclophane (0.40 g, 1.8 mmol), acenaphtenequinone (0.15 g, 0.81 mmol), and a small amount of acetic acid in EtOH (35 mL) was heated for 35 h under reflux. After removal of the solvent, purification by silica gel column (hexane/CH_2_Cl_2_, 2:1; R_f_ = 0.3) yielded ligand **L1** as an orange solid (0.26 g, 0.43 mmol, 53%). The ^1^H and ^13^C{^1^H} NMR spectra indicated the presence of conformation isomer whose structural details were not clarified. The obtained ligand was used for preparation of the complex directly. Anal. Calcd for C_44_H_36_N_2_: C 89.15; H 6.12; N 4.73. Found C 89.28; H 6.12, N 4.60.

### 4.3. Preparation of Optically Active Ligand ***L1***

A mixture of (*R*)-(–)-amino[2.2]paracyclophane (50 mg, 0.22 mmol) [64,65,66,67,68] and acetonaphtequinone (19 mg, 0.10 mmol) and a small amount of acetic acid in EtOH was heated for 24 h under reflux. Purification by alumina column (hexane/CH_2_Cl_2_, 2:1; R_f_ = 0.3) yielded ligand **L1** as an orange solid (34 mg, 0.56 mmol, 50%). The ^1^H and ^13^C{^1^H} NMR spectra are identical with the compound formed from racemic starting materials. Anal. Calcd for C_44_H_36_N_2__·_0.3H_2_O: C 88.35; H 6.17; N 4.68. Found C 88.25; H 5.98, N 4.65.

### 4.4. Preparation of NiBr_2_(***L1***)

A mixture of NiBr_2_(dme) (dme: 1,2-dimethoxyethane) (120 mg, 0.39 mmol) and (*rac*)-**L1** (240 mg, 0.41 mmol) in Et_2_O was stirred for 24 h at room temperature. The resulted solid was obtained by filtration, washed with Et_2_O to yield NiBr_2_(**L1**) as an dark brown solid (280 mg, 0.34 mmol, 96%). Anal. Calcd for C_44_H_36_N_2_Br_2_Ni: C 65.14; H 4.47; N 3.45. Found C 65.39; H 4.50, N 3.29. The reaction of (*R,R*)-**L1** with NiBr_2_(dme) was carried out analogously.

### 4.5. X-ray Crystallography of NiBr_2_(***L1***)

Single crystals of NiBr_2_(**L1**)·(C_2_H_4_Cl_2_) suited to X-ray diffraction study were obtained by recrystallization from 1,2-dichloroethane–Et_2_O, and mounted on MicroMounts (MiTeGen). The crystallographic data were collected on a Bruker SMART APEXII ULTRA/CCD diffractometer equipped with monochromated Mo Kα radiation (λ = 0.71073 Å). Calculations were carried out using the program package Olex2 [69]. Crystallographic data have been deposited with the Cambridge Crystallographic Data Centre: deposition number CCDC-2076633, which can be obtained free of charge via http://www.ccdc.cam.ac.uk/conts/retrieving.html.

### 4.6. Preparation of NiBr_2_(***L2***)

A mixture of NiBr_2_ (200 mg, 0.90 mmol), 2,5-dimethylaniline (0.26 mL, 2.2 mmol), and acetonaphthenequinone (180 mg, 1.00 mmol) was dissolved in acetic acid (5 mL) at 80 °C. After heating for 1 h at the temperature, the resulted solid was collected by filtration, washed with acetic acid and then Et_2_O, and dried in vacuo to give NiBr_2_(**L2**) as a yellow brown solid (540 mg, 0.89 mmol, 99%). Anal. Calcd for C_28_H_24_N_2_Br_2_Ni·0.5H_2_O: C 54.59; H 4.09; N 4.55. Found C 54.47; H 4.25, N 4.38.

### 4.7. Oligomerization

#### 4.7.1. Oligomerization of Ethylene

To a 25 mL Schlenk flask containing NiBr_2_(**L1**) (0.10 mmol) under nitrogen atmosphere was added dried toluene (10 mL) and naphthalene (64 mg, internal standard). The system was degassed by two freeze-thaw cycles. The flask was connected to a balloon filled with ethylene (1 atm), and MAO solution ([Al]/[Ni] = 300) was added to the mixture through septum. The reaction was conducted in a thermostated bath. A part of the product was extracted from the system by a syringe and analyzed by ^1^H NMR and GLC.

#### 4.7.2. Oligomerization of 1-Hexene

To a 25 mL Schlenk flask containing NiBr_2_(**L1**) (0.10 mmol) under nitrogen atmosphere was added dried toluene (1.5 mL) and a hexane solution of naphthalene (internal standard). The system was degassed by two freeze-thaw cycles, and the flask was filled with nitrogen. A hexane solution of MAO ([Al]/[Ni] = 300) was added through septum, and the reaction was carried out in a thermostated bath. A part of the product was extracted from the mixture, and analyzed by^1^H NMR and GLC.

#### 4.7.3. Co-Dimerization of Ethylene and 1-Hexene

A toluene solution of MMAO was evacuated to remove the solvent, and the remaininig MMAO was dissolved in pentane. To a 25 mL Schlenk flask containing NiBr_2_(**L1**) (0.10 mmol) under nitrogen atmosphere was added a pentane (5 mL) solution of 1-hexene and naphthalene (internal standard). The flask was connected to a balloon filled with ethylene (1 atm). The pentane solution of MMAO was added to the system via a syringe through septum. The reaction was carried out in a thermostatted bath, and a part of the product was extracted from the solution via a syringe.

## Figures and Tables

**Table 1:** Ethylene oligomerization catalyzed by NiBr_2_(**L1**) ^a^.

Entry	Catalyst	Conditions		Products/mmol			C4TOF/h^−1 b^
		Temp/°C	Time/h	1-Butene	2-Butene	Hexenes	
1	NiBr_2_(**L1**)	10	3	0.32	0.11	0.00	29
2	NiBr_2_(**L1**)	10	6	0.85	0.90	0.00	58
3	NiBr_2_(**L1**)	25	1	0.15	0.05	0.01	40
4	NiBr_2_(**L1**)	25	3	0.67	0.63	0.10	86
5	NiBr_2_(**L1**)	25	6	0.98	2.78	0.75	126
6	NiBr_2_(**L1**)	25	8	0.99	3.75	1.10	124
7	NiBr_2_(**L2**)	25	1	– ^c^	– ^c^	– ^c^	– ^c^
8	NiBr_2_(**L1**)	50	3	0.12	0.18	0.00	20
9	NiBr_2_(**L1**)	50	6	0.20	0.34	0.00	18

^a^ Conditions: [Ni] 0.010 mmol, MAO co-catalyst ([Al]/[Ni] = 300), toluene 1 mL, ethylene 1 atm. ^b^ TOF [mol (2 × C4)][mol cat.]^−1^ h^−1^. ^c^ The product was polyethylene with *M*_n_ = 1000 (GPC).

**Table 2:** Oligomerization of 1-hexene catalyzed by NiBr_2_(**L1**) ^a^.

Entry	Co-Catalyst ^b^	Conditions		Products (%)			C12,C18 TOF/h^−1^
		Temp/°C	Time/h	2-Hexene	C12	C18	
1	MAO (300)	10	6	34	2.9	6.6	4.8
2	MAO (300)	10	24	79	9.5	8.6	2.3
3	MAO (50)	25	24	66	2.6	17	2.5
4	MAO (300)	25	6	56	7.3	13	10.2
5	MAO (300)	25	24	62	11	18	3.6
6	MAO (1000)	25	24	21	2.1	18	2.5
7	MAO (300)	35	0.5	33	4.6	0.0	28
8	MAO (300)	35	6	54	16	5.6	11
9	MAO (300)	35	24	45	22	28	6.3
10	MAO (300)	50	0.5	49	7.2	3.0	61
11	MAO (300)	50	6	40	11	34	23
12	MAO (300)	50	24	23	12	57	8.6
13	MMAO (300)	25	6	37	6.8	18	12
14	AlMe_3_ (300)	50	1	98	0.0	0.0	0.0
15	Et_2_AlCl (300)	50	1	95	0.0	0.0	0.0

^a^ Conditions: catalyst NiBr_2_(**L1**), [Ni] 0.010 mmol, [1-hexene]/[Ni] = 300, solvent toluene (1.5 mL). ^b^ [Al]/[Ni] is shown in parenthesis.

## Data Availability

Data is contained within this article.
